# A Pilot Study of Implementing Diabetic Retinopathy Screening in the Oslo Region, Norway: Baseline Results

**DOI:** 10.3390/biomedicines11041222

**Published:** 2023-04-19

**Authors:** Ellen Steffenssen Sauesund, Øystein Kalsnes Jørstad, Cathrine Brunborg, Morten Carstens Moe, Maja Gran Erke, Dag Sigurd Fosmark, Goran Petrovski

**Affiliations:** 1 Department of Ophthalmology, Oslo University Hospital, 0450 Oslo, Norway; 2 Center for Eye Research and Innovative Diagnostics, Department of Ophthalmology, Institute of Clinical Medicine, Faculty of Medicine, University of Oslo, 0450 Oslo, Norway; 3 Oslo Centre for Biostatistics and Epidemiology, Research Support Services, Oslo University Hospital, 0450 Oslo, Norway

**Keywords:** diabetic retinopathy, diabetes mellitus, screening, Norway

## Abstract

Purpose: to gain insight into the baseline parameters of a population with diabetes mellitus (DM) included in a pilot diabetic retinopathy (DR) screening program at Oslo University Hospital (OUH), Norway. Methods: This was a cross-sectional study of a cohort of adult patients (≥18 years) with type 1 or 2 DM (T1D and T2D). We measured the best-corrected visual acuity (BCVA), blood pressure (BP), heart rate (HR), intraocular pressure (IOP), height and weight. We also collected HbA1c, total serum cholesterol and urine-albumin, -creatinine and -albumin-to-creatinine ratio (ACR), as well as socio-demographic parameters, medications and previous screening history. We obtained color fundus photographs, which were graded by two experienced ophthalmologists according to the International Clinical Disease Severity Scale for DR. Results: The study included 180 eyes of 90 patients: 12 patients (13.3%) had T1D and 78 (86.7%) had T2D. In the T1D group, 5 patients (41.7%) had no DR, and 7 (58.3%) had some degree of DR. In the T2D group, 60 patients (76.9%) had no DR, and 18 (23.1%) had some degree of DR. None of the patients had proliferative DR. Of the 43 patients not newly diagnosed (time of diagnosis > 5 years for T1D and >1 years for T2D), 37.5% of the T1D patients and 5.7% of the T2D patients had previously undergone regular screening. Univariate analyses found for the whole cohort significant associations between DR and age, HbA1c, urine albumin-to-creatinine ratio, body mass index (BMI) and duration of DM. For the T2D group alone, there were significant associations between DR and HbA1c, BMI, urine creatinine, urine albumin-to-creatinine ratio and duration of DM. The analysis also showed three times higher odds for DR in the T1D group than the T2D group. Conclusions: This study underscores the need for implementing a systematic DR screening program in the Oslo region, Norway, to better reach out to patients with DM and improve their screening adherence. Timely and proper treatment can prevent or mitigate vision loss and improve the prognosis. A considerable number of patients were referred from general practitioners for not being followed by an ophthalmologist.Among patients not newly diagnosed with DM, 62.8% had never had an eye exam, and the duration of DM for these patients was up to 18 years (median: 8 years).

## 1. Introduction

Diabetic retinopathy (DR) is a frequent complication of diabetes mellitus (DM) [1]. Still, it often remains asymptomatic until advanced disease has developed, which is one of the reasons for systematic DR screening programs. The retinal microvasculopathy of DM causes ischemia and hypoxia, the latter leading to the release of vascular endothelial growth factor (VEGF), which is able to induce both new vessel (NV) formation and edema. These NVs may act as a source of low-grade bleeding and leakage of lipoproteins and fluid, infiltrating and changing the functional architecture of the retina. As the natural history of DR is well known, further sight-threatening lesions may ensue, such as hemorrhages, macular edema and more severely increased intraocular pressure (IOP) due to the fact of neovascular glaucoma and tractional retinal detachment.

DR is one of the leading causes of global blindness in those aged 50 years and older, and DR-related complications represent a main cause of impaired vision in patients aged 25–74 years [2,3]. DR is responsible for a severe economic burden and reduced quality of life [2,4]. In a pooled analysis comparing data from 35 populations [5], sight-threatening DR (STDR) (i.e., presence of preproliferative or proliferative DR, maculopathy or evidence of photocoagulation treatment) was estimated to affect 10.2% of DM patients. Data from England and Wales show a vision-saving effect of implementing a comprehensive program for regular retinal examinations. During a 10-year observation period, the proportion of newly blind people due to the fact of DM was reduced by approximately 20% [6]. Hence, there are reasons to assume that such an impact could be transferable to Norway and that a more personalized program for regular retinal examinations may further reduce the proportion of newly blind people. Still, the screening rate in Norway is only approximately 62% [7]. Regular retinal examinations to detect DR at an early stage is also known to be cost effective [8].

For people with a known DM for more than 20 years, 76% develop some degree of DR [5]. Other studies have shown that approximately 20% and 37% of patients with newly diagnosed T2D have already developed DR [9,10]. Accordingly, guidelines recommend the initiation of DR screening to begin at the time of T2D diagnosis.

The global prevalence of DM (age: 20–79 years) in 2021 was approximately 537 million (61 million in Europe alone), and the number is estimated to rise to 643 million (67 million in Europe) by 2030 and 783 million (69 million in Europe) by 2045 [11]. The National Diabetes registry for adults in Norway is incomplete (25% of GPs reporting), but it is estimated primarily by the Norwegian Prescription Database that approximately 316,000–345,000 persons (5.9–6.4% of the population) have DM, of whom 60,000 are undiagnosed [12]. Moreover, approximately 90% of DM patients in Norway are estimated to have T2D [13]. Data from The Norwegian Prescription Database show that 4.1% used blood-glucose-lowering drugs in 2020, representing a 70.3% increase from 2004 and a 32.1% increase from 2010 [14]. Because of the increasing prevalence of DM [11], it can be assumed that the prevalence of DR will further increase.

The living conditions in Norway have been ranked as high by a United Nations’ Human Development Report [15], albeit a systematic national DR screening program as of yet has not been implemented. There is a lack of knowledge concerning the DM and DR prevalence, as well as risk factors in people with DM, in Oslo, Norway. This was incentive for a pilot study for baseline insight as a step towards establishing an optimal DR screening program.

## 2. Materials and Methods

This was a cross-sectional study of a cohort of adult patients (≥18 years of age) with T1D or T2D belonging to the region of OUH, Oslo, Norway. The patients were mainly referred from general practitioners after they received information concerning the project (12 patients were referred from another healthcare institution). The general practitioners were invited to refer patients without a known treatment-dependent DR and who were not already followed by an ophthalmologist. A total of 90 patients (180 eyes) were enrolled, and written informed consent was obtained from all participants. The patients were included in the period from December 2019 to January 2021. The Regional Committee for Medical and Health Research Ethics concluded that the project was outside the remit of the Norwegian Health Research Act (reference: 28857). The Institutional Data Protection Officer at OUH approved the study (reference: 20/00571).

The study took place at the Department of Ophthalmology, OUH. Best-corrected visual acuity (BCVA) was assessed using the Clear Chart 2 (Reichert Technologies, Depew, NY, USA) digital acuity test, which displays 5 letter optotypes per line and a logarithm of the minimal angle of resolution (logMAR) line size progression (i.e., each letter has a score of 0.02 logMAR). After one minute of rest, blood pressure (BP) and heart rate were measured in the left overarm using a calibrated automatic BP monitor (Riester, ri-champion N Automated Blood Pressure Monitor, Jungingen, Germany). Hypertension (HT) was defined as hypertension grade 2 according to the American Heart Association: systolic at least 140 or diastolic at least 90 mm Hg (≥140/90 mmHg). We measured the intraocular pressure (IOP) using an iCare ic 100 tonometer (Icare Finland Oy, Vantaa, Finland). Mean ocular perfusion pressure (MOPP) was calculated as two-thirds of the systemic mean arterial pressure (MAP) minus the IOP [16]. The MAP = diastolic blood pressure (DBP) + one-third (systolic blood pressure (SBP)−DBP). Prior to the imaging and fundus examination, the pupils were dilated with topical tropicamide 0.5%.

If not available from the referral, the following laboratory tests were performed at the department: HbA1c, total serum cholesterol and urine-albumin, -creatinine and -albumin-to-creatinine ratio (ACR).

Color fundus photography was performed using the CLARUS^TM^ 700, Zeiss (Carl Zeiss Meditec AG, Jena, Germany). Fovea- and optic disc-centered images were obtained, both with a 133° field of view.

The fundus images were graded according to the International Clinical Disease Severity Scale for DR [17]: no DR, mild non-proliferative DR (NPDR), moderate NPDR, severe NPDR or proliferative DR. Both eyes were graded through consensus between two experienced ophthalmologists (E.S.S. and D.F.); the eye with the more severe retinopathy defined the individual grade.

Diabetic maculopathy based on fundus photography was classified as follows: no diabetic maculopathy (0), presence of microaneurysm(s) within 1 disc diameter from the foveola (1) and hard exudate(s) within 1 disc diameter from the foveola (2).

Socio-demographic parameters, such as gender, age, type of DM, duration of DM, use of tobacco and alcohol, body weight and body height (to determine BMI), were registered. We also documented medication history (type of DM medication, duration of insulin treatment and use of cholesterol lowering- and antihypertensive drugs) and previous history regarding DR screening.

We used the Mann–Whitney U test to investigate the differences between two groups for continuous variables and the Chi-square or Fisher’s exact test (for small cell counts) to detect associations among categorical variables. All tests were two-sided, and a 5% significance level was defined. To investigate the factors associated with retinopathy, a generalized estimating equation (GEE) analysis was applied to adjust for intra-individual correlation (since both eyes of each individual were included). We used IBM SPSS Statistics 28.0 (IBM Corp., Armonk, NY, USA) for the data analyses.

## 3. Results

Altogether 180 eyes of 90 patients (61 males (67.8%) and 29 females (32.2%)) were included in the study.

**Figure 1:**
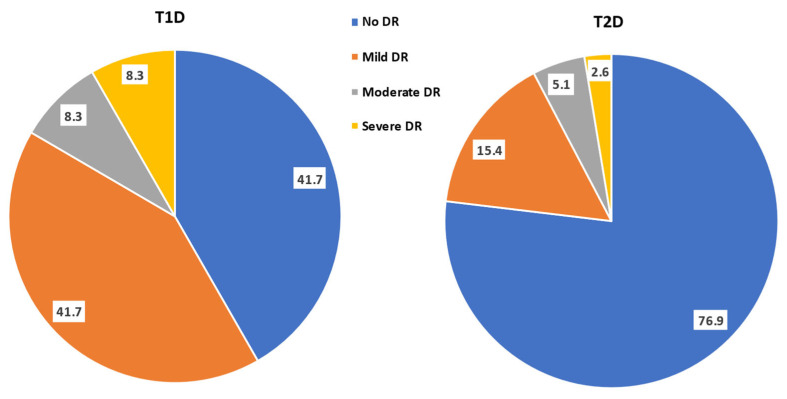
Pie chart (percentage frequency distribution) of the grades of diabetic retinopathy in the studied population having type 1 and type 2 diabetes mellitus (T1D and T2D).

The sizes of the T1D and T2D populations amounted to 12 (13.3%, 95% CI: 7.8, 21.9) and 78 (86.7%, 95% CI: 78.1, 92.2), respectively (Figure 1). There were 5 (41.7%, 95% CI: 19.3, 68.1) and 60 (76.9%, 95% CI: 83.2, 96.7) patients with no DR in the T1D versus the T2D group, while 7 (58.3%, 95% CI: 14.3, 47.6) and 18 (23.1%, 95% CI: 52.4, 85.7) patients had DR in the T1D and T2D groups, respectively. The distribution of the different grades of retinopathy were 5 (41.7%, 95% CI: 19.3, 68.1) and 12 (15.4%, 95% CI: 9.0, 25.0) with mild DR, 1 (8.3%,95% CI: 1.5, 35.4) and 4 (5.1%, 95% CI: 2.0, 12.5) with moderate DR, 1 (8.3%, 95% CI: 1.5, 35.4) and 2 (2.6%, 95% CI: 0.7, 8.9) with severe retinopathy in the T1D and T2D group, respectively; 0 had proliferative DR in either group.

Table 1 shows the demographic characteristics of the studied cohort where 12 (13.3%) had T1D and 78 (86.7%) had T2D. Among the T1D patients, 5 (41.7%) had no DR and 7 (58.3%) had DR, whereas 60 (76.9%) of the T2D patients had no DR and 18 (23.1%) had DR.

For the T1D group, the median age was 38 (IQR 30.5, 54.0) for patients without DR and 31 (IQR 22.0, 43.0) for patients with DR. For the T2D group, the median age was 54 (IQR 44.8, 62.0) for patients without DR and 51.5 (IQR 37.3, 58.0) for patients with DR.

The median number of years since diagnosis of DM for the T1D patients having no DR was 4.0 (IQR: 0, 8.0) and having DR was 17.0 (IQR: 15.0, 23.0), while for the T2D patients it was 1.0 (IQR: 0, 3.8) and 11.0 (IQR: 5.8, 14.3), respectively.

Two T1D patients had obesity and DR (28.6%) (body mass index (BMI) ≥25), and two had obesity and no DR (40.0%); in the T2D group, obesity and DR was present in thirteen (72.2%) patients and obesity and no DR in forty-nine (81.7%) patients.

Table 2 shows the screening history of our study cohort. A total of 47 (52.2%) patients were newly diagnosed with DM (e.g., time since diagnosis was <5 years for patients with T1D and <1 year for patients with T2D): 4 (8.5%) T1D and 43 (91.5%) T2D. In the T1D group, 3 (75.0%) had non-DR and 1 (25.0%) had DR, while in the T2D group, 40 (93.0%) had non-DR and 3 (7.0%) had DR. Overall, 8.5% of the patients who were newly diagnosed with DM had DR.

A total of 43 (47.8%) patients were not newly diagnosed with DM (e.g., time since diagnosis > 5 years for patients with T1D and >1 year for patients with T2D); 8 (18.6%) of these patients had T1D and 35 (81.4%) had T2D. In the T1D group, 2 patients (25.0%) had no DR and 6 (75.0%) had DR, while in the T2D group, 20 patients (57%) had no DR and 15 (43%) had DR. Overall, 48.8% of the patients who were not newly diagnosed with DM had DR.

Of the 43 patients not newly diagnosed with DM, 16 (37.2%) had previously undertaken an eye exam. For 11 (68.8%) of the patients who had an eye exam, it was more than 2 years, with a maximum of 10 years since the last eye exam.

Twenty-seven (62.8%) patients not newly diagnosed with DM had not undertaken an eye exam (only T2D patients), and the duration of DM for these patients varied from 2 to 18 years (median 8 years); twelve (44.4%) of these patients had had DM for > 10 years.

With regard to antidiabetic drugs (Table 3), 9 out of 12 patients (75.0%) with TD1 used insulin, and the duration of insulin treatment ranged from 0 to 34 years; 8 out of 78 patients (10.3%) with T2D used insulin, and the duration of treatment ranged from 0 to 18 years; 3 out of 12 patients (25.0%) in the T1D group and 58 out of 78 patients (74.4%) in the T2D group used other antidiabetic medications (OAMs). Both insulin and OAMs were used by 4 out of 78 patients (5.1%) in the T2D group but none in the T1D group. None of the patients with T1D used glucagon-like peptide 1 analogues (GLP-1 analogues). In the T2D group, three patients (17.6%) with DR and four patients (7.8%) without DR used GLP agonists. None of the T1D patients used cholesterol-lowering medications, while 31 of the T2D patients (22 without DR (36.7%) and 9 with DR (50%)) used cholesterol medications. One patient in the T1D group having DR (14.1%) was on antihypertensive medication and in the T2D group forty patients (thirty-three without DR (55.0%) and seven with DR (38.9%)), respectively.

Table 4 shows the odds ratio for DR in the studied population (T1D and T2D) and in the T2D population alone.

The univariate analysis found for the whole cohort a significant association between DR and the following parameters: age increase of 1 year showed a 2% decrease (*p* = 0.002) in the odds ratio for DR; an increase of 11 mmol/mol HbA1c showed a 19% increase (*p* = 0.003) a 1-unit increase in the urine albumin-to-creatinine ratio showed a 1% increase (*p* = 0.008); a 1-unit increase in BMI showed a 6% decrease (*p* < 0.001); and a 1-year increase in DM duration showed a 13% increase (*p* < 0.001) in the odds ratio for DR.

The same analyses for the T2D population alone found a significant association between DR and the following parameters: an HbA1c increase of 11 mmol/mol showed a 31% increase (*p* < 0.001); an increase of 1 unit of BMI showed an 8% decrease (*p* = 0.001); and an increase of 1 year in DM duration showed an 11% increase (*p* < 0.001) in the odds ratio for DR.

There was a significant difference (*p* < 0.001) in the odds ratio for DR in the T1D versus the T2D group, with three times higher odds (OR 3.05) for DR in the T1D group.

Table 5 shows the presence of diabetic maculopathy based on fundus photos in the study cohort. None of the patients without DR had diabetic maculopathy; 43 eyes (23.9%) had DR, and in 27 (62.8%) of these eyes, diabetic maculopathy was also present.

In the T1D group, DR was found in 14 (58.3%) eyes, and 9 (64.3%) of these eyes had diabetic maculopathy based on fundus photos. All eyes with diabetic maculopathy had MA within 1 DD from the foveola, and one of these eyes (11.1%) also had hard exudates.

In the T2D group, DR was found in 29 (18.6%) eyes, and 18 (62.1%) of these eyes had diabetic maculopathy based on fundus photos. All eyes with diabetic maculopathy had MA within 1 DD from the foveola, and three of these eyes (16.7%) also had hard exudates.

## 4. Discussion

The present pilot study provides us baseline screening information concerning the population with DM in the Oslo region, Norway. This is valuable for planning and establishing an optimal systematic screening system in this area of the country, and further, to establish a national screening system in Norway. The results from our pilot study demonstrate a lack of ophthalmological examinations and follow-ups for DM patients and the need for implementing a proper and systematic DR screening.

The majority of the 90 patients (180 eyes) recruited in our study came from general practitioners in the Oslo region, Norway. Approximately half of the patients were newly diagnosed with DM (time since diagnosis ≤5 years for patients with T1D and ≤1 year for patients with T2D). Two patients with T1D in this category had late autoimmune diabetes in the adults (LADA). Considering the group of patients without a newly diagnosed DM, nearly two-thirds of them had never had an eye exam performed. The duration time of DM for these patients varied from 2 to 18 years (median: 8 years), and 12 patients had DM > 10 years. Only 37% of the patients not newly diagnosed had a previous eye exam, and little over two-thirds of these patients had more than 2 years since their last eye exam. Implementing a proper and systematic DR screening in our region is needed for better reaching out to patients with DM, to improve timely follow-up and thereby reduce DR progression and vision loss.

The prevalence of any DR in patients with T1D and T2D were 58.3% (95% CI: 14.3, 47.6) and 23.1% (95% CI: 52.4, 85.7), respectively, which is similar to other population-based studies [10,18,19]. It is challenging, however, to compare the prevalence of DR among studies, since there are differences between grading protocols and populations. A Norwegian study, from 2012, by Kilstad et al. [7] found the prevalence of any DR in T1D to be 66%, which is higher than in our study, whereas the prevalence for any DR in T2D was 24%, which is in line with our study. Established treatment-dependent DR was an exclusion criterion in this study, and approximately half of the patients were newly diagnosed with DM, which could have affected the results obtained compared to other studies. In particular, the high proportion of newly diagnosed patients can be part of the explanation for why there were no cases of proliferative DR and few cases of severe DR. Still, another Norwegian study, from 2008, by Sundling et al. [20] reported an even lower prevalence of DR. However, this was a questionnaire-based survey study.

In other parts of Europe, Looker et al. [10] found the prevalence of DR in a population with newly diagnosed T2D in Scotland to be 19.3%. According to the Liverpool Diabetic Eye Study [18], the prevalence of DR was 45.7% and 25.3% for T1D and T2D, respectively, and the United Kingdom Prospective study (UKPDS) [21] found a prevalence of 37% for DR at diagnosis in a cohort of patients with T2D. In a cross-sectional study from Wales [19], the prevalence of any DR for T1D and T2D was 56.3% and 30.9%, respectively.

By comparison, a recent systematic review and meta-analysis showed that the prevalence of DR in patients with DM during 2020 was highest in the Middle East and North Africa (32.90%), North America and the Caribbean (33.30%), moderate in Southeast Asia (16.99%) and Western Pacific (19.20%) and lowest in South and Central America (13.37%) [22]. In China, the reported prevalence of DR in patients with DM in a relatively small cohort carried out between 2018 and 2019 was also high (40%) [23].

It is well known that the duration of DM is a major risk factor for DR [18,19,24,25,26,27,28], as also demonstrated by our study. Notably, each one-year increase in the duration of DM showed a 13% increase in the OR for DR (OR 1.13; 95% CI 1.09, 1.17; *p* < 0.001), and in the T2D group alone an increase of 1 year in duration of diabetes showed an 11% increase in the OR for DR (OR 1.11; 95% CI 1.07, 1.16; *p* < 0.001). In our cohort of patients, an age increase of 1 year showed a 2% decrease in the OR for DR (OR 0.98; 95% CI 0.96, 0.99; *p* = 0.002). This could be related to the younger age of the studied population in the T1D group and the higher number of patients with DR in this group itself. Other studies have also shown an association of DR with younger age [29,30]. No significant association was found between DR and age in the T2D population alone in our cohort.

For both groups T1D and T2D (OR 0.94; 95% CI 0.90, 0.97; *p* < 0.001) and for T2D alone (OR 0.92; 95% CI 0.88, 0.97; *p* = 0.001), there was an inverse association for DR with increased BMI, in accordance with other studies [31,32].

An increase of 11 mmol/mol HbA1c showed a 19% increase in the OR for DR for the whole cohort (OR 1.19; 95% CI 1.06, 1.34; *p* = 0.003) and a 31% increase for the T2D population alone (OR 1.31; 95% CI 1.15, 1.50; *p* < 0.001). This higher risk for DR with the increase in HbA1c is in line with multiple studies from several countries, both for T2D [21,27] and T1D [27,33,34,35].

For the urine creatinine level, a significant inverse association with DR was found in the T2D group (OR 0.95; 95% CI 0.91, 0.997; *p* = 0.036), which is in accordance with a population-based study of patients with T2D [36]. In both this study and others [37,38], the urine albumin-to-creatinine ratio showed a positive association with DR, similar to our cohort of both T1D and T2D grouped together (OR 1.01; 95% CI 1.003, 1.02; *p* = 0.008) but also in the T2D group alone (OR 1.01; 95% CI 1.002, 1.02; *p* = 0.016).

T1D is a known risk factor for DR [39,40], and we also found three times higher OR (OR 3.05; 95% CI 1.75, 5.32; *p* < 0.001) for DR in the T1D group compared to the T2D group. It needs to be taken into consideration the small number of T1D patients in our study cohort, which is a limitation of our study.

No significant association between DR and HT was found in our population; meanwhile, several other studies have found a positive association with HT [21,34,35,41]. Gender, total plasma cholesterol, urine albumin, microalbuminuria, HT, SBT, DBT, MAP, smoking, use of smokeless tobacco, alcohol, VA, IOP, BCVA and MOPP also did not show a significant association with DR.

Three-quarters of the patients with T1D and one-tenth of the patients with T2D were on insulin treatment. The three patients in two newly diagnosed patients with LADA and one not newly diagnosed patient with MODY3. In other population-based studies [7,42], 17–18% of the patients with T2D were on insulin treatment. The duration of the insulin treatment was the highest in the T1D group with DR, and the median duration of the insulin treatment in this group was more than double the time found in the T2D group with DR. In our pilot study, 67.8% used OAM, 4.4% were on insulin and OAM treatment and 7.8% were on GLP analogues. Regarding the percent of patients on GLP analogues, this is in accordance with a cohort study from the US, which included over a million patients with DM [43].

Diabetic maculopathy was found in more than one-third of the eyes examined in the T1D group and little over one-tenth of the eyes in the T2D group, with a total prevalence of 15%. The duration time of DM was 17 years in the T1D group with DR versus 11 years in the T2D group with DR. This and the limited number of patients in the T1D group can, to some extent, explain the impact on the divergent results obtained in the two groups. Compared to another cross-sectional study [44] of patients with T1D and T2D, the prevalence of diabetic maculopathy was lower in our study. The population included in the other study presented a longer duration of DM, patients and not eyes were observed, and the grading protocol was different from ours, which surely can affect the results.

## 5. Conclusions

This pilot study provides valuable baseline screening information concerning a DM population in the Oslo region, Norway. The results demonstrate a lack of ophthalmological examinations and follow-up for a considerable number of patients. Among the patients not newly diagnosed with DM, 62.8% had never had an eye exam; it was > 2 years since the last eye exam for 68.8% of the patients who had a previous eye exam. Implementing a systematic DR screening program is needed to better reach out to patients with DM and improve their screening adherence, thereby provide timely and proper treatment.

## Figures and Tables

**Table 1:** Demographics of the studied population.

	T1D		T2D	
	No DR, *n* = 5	DR, *n* = 7	No DR, *n* = 60	DR, *n* = 18
**Gender**				
Male, *n* (%)	3 (60.0)	5 (71.4)	39 (65.0)	14 (77.8)
Female, *n* (%)	2 (40.0)	2 (28.6)	21 (35.0)	4 (22.2)
**Age, (yrs, median (IQR))**	38.0 (30.5, 54.0)	31.0 (22.0, 43.0)	54.0 (44.8, 62.0)	51.5 (37.3, 58.0)
**Years since diagnosis, (median (IQR))**	4.0 (0, 8.0)	17.0 (15.0, 23.0)	1.0 (0, 3.8)	11 (5.8, 14.3)
**BMI > 25, *n* (%)**	2 (40.0)	2 (28.6)	49 (81.7)	13 (72.2)

**Table 2:** Screening history of the studied population.

	T1D		T2D		Total, *n* = 90
	No DR, *n* = 5	DR, *n* = 7	No DR, *n* = 60	DR, *n* = 18	
Patients newly diagnosed with DM **, *n* (%)	3 * (60.0)	1 (14.3)	40 (66.7)	3 (16.7)	47 (52.2)
Patients not newly diagnosed with DM ***, *n* (%)	2 (40.0)	6 (85.7)	20 (33.3)	15 (83.3)	43 (47.8)
Previous eye exam, *n* (%) of patients not newly diagnosed with DM	2 (100)	6 (100)	2 (10.0)	6 (40.0)	16 (37.2)
>2 years since last eye exam, *n* (%) of patients not newly diagnosed with DM	1 (50.0)	4 (66.7)	1 (50.0)	5 (83.3)	11 (68.8)
Patients followed-up as recommended, *n* (%) of patients not newly diagnosed with DM	1 (50.0)	2 (33.3)	1 (2.3)	1 (6.7)	5 (11.6)

* Two patients with T1D who were newly diagnosed had LADA. ** First visit within 1 year from diagnosis for patients with T2D and within 5 years for patients with T1D. *** First visit >5 years for patients with T1D and >1 year from diagnosis of patients with T2D.

**Table 3:** Diabetes, cholesterol-lowering and antihypertensive medications used in the studied population.

	T1D		T2D	
	No DR, *n* = 5	DR, *n* = 7	No DR, *n* = 60	DR, *n* = 18
**Diabetes medication**				
Insulin, *n* (%)	3 (60.0)	6 (85.7)	4 (6.7)	4 (22.2)
Years of insulin, median (range)	8.0 (0.0–8.0)	16.5 (1.0–34.0)	0 (0.0–11.0)	7.0 (3.0–18.0)
OAM * *n* (%)	2 ** (40.0)	1 ** (14.3)	43 (71.7)	15 (83.3)
OAM + insulin, *n* (%)	0 (0)	0 (0)	2 (3.3)	2 (11.1)
GLP analogues ***, *n* (%)	0 (0)	0 (0)	4 (7.8)	3 (17.6)
**Cholesterol-lowering medications, *n* (%)**	0 (0)	0 (0)	22 (36.7)	9 (50.0)
**Antihypertensive medications, *n* (%)**	0 (0)	1(14.3)	33 (55.0)	7 (38.9)

* OAM: other antidiabetic medication. ** Of the 3 patients in the T1D group not using insulin, 2 were newly diagnosed with LADA (2) and 1 not newly diagnosed had MODY3 (1). *** Glucagon-like peptide-1 analogues.

**Table 4:** Univariate analyses of the different parameters of the studied population.

	Total (T1D and T2D)		Only T2D	
	OR (95% CI)	*p*-Value	OR (95% CI)	*p*-Value
Age per 1 year	0.98 (0.96, 0.99)	**0.002**	0.99 (0.97, 1.01)	0.283
Male gender	1.40 (0.89, 2.19)	0.144	1.49 (0.88, 2.53)	0.137
HbA1c per 11 mmol/mol	1.19 (1.06, 1.34)	**0.003**	1.31 (1.15, 1.50)	**<0.001**
Total cholesterol	0.91 (0.74, 1.10)	0.322	0.99 (0.80, 1.22)	0.900
Urine creatinine	0.98 (0.95, 1.01)	0.268	0.95 (0.91, 0.997)	**0.036**
Urine albumin	1.001 (0.9999, 1.001)	0.101	1.001 (0.9999, 1.002)	0.524
Urine album-to-creatinine ratio	1.01 (1.003, 1.02)	**0.008**	1.01 (1.002, 1.02)	**0.016**
Microalbuminuria	0.95 (0.58, 1.56)	0.835	0.99 (0.58, 1.71)	0.989
BMI	0.94 (0.90, 0.97)	**<0.001**	0.92 (0.88, 0.97)	**0.001**
Duration of diabetes per 1 year	1.13 (1.09, 1.17)	**<0.001**	1.11 (1.07, 1.16)	**<0.001**
HT *	1.01 (0.66, 1.55)	0.948	0.86 (0.53, 1.39)	0.533
SBP	0.99 (0.98, 1.002)	0.104	0.99 (0.98, 1.001)	0.355
DBP	0.99 (0.97, 1.01)	0.351	1.00 (0.98, 1.02)	0.962
MAP	0.99 (0.97, 1.01)	0.162	0.995 (0.98, 1.02)	0.645
Type DM (1 vs. 2)	3.05 (1.75, 5.32)	**<0.001**		
Smoke (yes/no)	1.12 (0.64, 1.96)	0.695	0.45 (0.17, 1.22)	0.118
Smokeless tobacco (yes/no)	0.62 (0.34, 1.14)	0.124	0.80 (0.43, 1.51)	0.496
Alcohol	0.99 (0.92, 1.07)	0.853	0.92 (0.83, 1.02)	0.093
BCVA	1.29 (0.32, 5.26)	0.721	3.24 (0.71, 14.76)	0.129
IOP	0.97 (0.91, 1.04)	0.363	0.98 (0.91, 1.06)	0.657
MOPP	0.98 (0.96, 1.01)	0.210	0.99 (0.96, 1.03)	0.717

Note: Four patients had urine parameters missing, while 1 patient had HbA1c missing. * SBP ≥ 140 mmHg and/or DBP ≥ 90 mmHg.

**Table 5:** Presence of diabetic maculopathy on fundus photo in the studied population.

	**T1D** **Number of Eyes = 24**		**T2D** **Number of Eyes = 156**		**Total Number of Eyes = 180**
	**Number of Eyes with DR = 14**		**Number of Eyes with DR = 29**		**Total Number of Eyes with DR = 43**
	**OD**	**OS**	**OD**	**OS**	
**Diabetic maculopathy, *n*** (%) of total eyes with DR in the same group	4 (28.6)	5 (35.7)	11 (37.9)	7 (24.1)	27 (62.8)
(1) Microaneurysm, *n* (%) of total eyes with DR and diabetic maculopathy in the same group	4 (100)	5 (100)	11(100)	7 (100)	27 (100)
(2) Hard exudates, *n* (%) of total eyes with DR and diabetic maculopathy in the same group	0	1 (0.2)	2 (18.2)	1 (14.3)	4(14.8)

## Data Availability

Data from the project can be requested directly from the corresponding author.
